# Investigation of aerobic training and electrical motivation impacts on body fat decrease

**Published:** 2015

**Authors:** E Sekhavati, A Nikseresht, M Najafian, S Taheri

**Affiliations:** * Larestan School of Medical Sciences, Larestan, Iran; ** Islamic Azad University, Jahrom Branch, Iran

**Keywords:** electrical stimulation, walking, body fat

## Abstract

**Goals:** Study and comparison between the effects of walking and the electrical motivation on body fat burning and weight loss.

**Methodology:** The samples were elected among the volunteer healthy females (20-40 years) with an index rate (20 ≤ BMI ≤ 30) provided that they had no regular active exercise activity and they were classified randomly to 2 teams in this quasi-experimental research. The electrical stimulation was used for one group in 1 year each week up to eight, and the period of 40-60 min per session on intestinal muscles. The plan of the group with aerobic exercise included 40-60 min walking with an intensity of the stored heart rate (50-70%) within the similar period. The parameters of BMI, rate of body fact, and LBM were measured at the start and at terminal of study on weight. The given data are investigated via using of t- independent and correlation t-test at level (p≤ 0.05).

The parameters of BMI, rate of body fact, and LBM were measured at the start and at terminal of study on weight.

**Findings:** Weight, Body Mass Index (BMI), and body fat percentage were significantly reduced and also the LBM parameter was enhanced in 2 teams.

**Conclusion:** Walking and electrical stimulation similarly caused to decrease in weight, BMI, body fat percentage, and enhance in lean body mass factor (LBM).

## Introduction

Today, obesity is one of the highest epidemic health issues in the universe and its incidence is going to increase [**5**,**15**]. The recent findings signify that inappropriate lifestyle and lesser daily activity cause an advance in the obesity predominance and particularly the abdominal fatness [**24**]. 

According to a study of WHO in 2005, about 1.6 milliard adults were overweight and it is anticipated that by 2015 this population were 2.3 milliard peoples via fatty and 700 million obese persons [**22**].

Likewise, based on another report, more than 60% of males and 50% of females in the world, except for South and East Asia, were overweight and fat. This frequency rate was estimated about obesity with a rate of 30 ≤ BM of 7% in eastern Asia and up to 36% in Canada for both female and male genders, and also 38-40% of females in Central Asia, North Africa, and South Africa [**13**].

Epidemics of obesity in Developing Countries like Iran have been also accompanied by a growing trend due to the rising level of welfare for people in two recent decades [**17**]. Pishdad (1996) reported a higher rate of obesity prevalence and particularly fatty and a lesser level of bodily actions in Iranian males. Similarly, Azadbakht et al. (2005) mentioned the ratio of prevalence of public obesity as 29% in 4’164 males in Tehran city. 

It has been known until today that obesity is an free danger portion in continuous illnesses and may increase the danger of untimely destruction [**12**,**14**] so that the risk of reduced lifetime in obese people is 40% higher than of the other persons and this rate reaches to 70% in a person with excessive obesity [**9**]. Also concurrently it was characterized that the rising risk of suffering from regenerative and chronic diseases is accompanied by obesity hence the diseases of hypertension, blood fat, arthrosclerosis of walls in coronary vessels, arthritis, and also joints contractures and obstructive consumptive disorders are straight associated with obesity, and the obese people suffer from various deficiencies in metabolism of carbohydrates and they gradually suffer from diabetes type-II [**18**, **27**]. 

While the fat may be accumulated in any point of body, the related risks depend on the region in which the fat has been accumulated [**25**], so that according to the viewpoint of researchers, the over-storing of fat in the abdominal point may threaten the health and importance of the study in this subject being revealed, when we know that abdominal obesity is much more risky than the collection of lipids in the another points of the body; there was a clear association among the rate of abdominal fat and the various diseases and it is followed by premature mortality [**12**,**14**-**16**,**19**,**26**]. 

The existing significant relationship among the ratio of waist circumference and cardiovascular danger parameter and diabetes suggests the fact that the obesity in the central part of body (abdomen) is assumed as a free hazard parameter in cardiovascular problems [**3**,**5**,**8**,**10**]. Thus, recently, the central obesity has been used as a main predictor for the risk of suffering from cardiovascular diseases [**13**].

The epidemics of abdominal obesity have been in turn noticeable during recent years so that the rate of abdominal obesity has been stated to be of 29% in men and 48% among females. Nonetheless, this statistical rate among Caucasian people is of 56% and 71% for men and women, consequently [**13**]. 

Therefore, the scientists have employed several methods in their pursuit to find an appropriate technique to reduce fat and particularly abdominal lipid in this field including massage, low-calories diet, drug and hormonal methods, slimming belts, acupuncture, and liposuction surgery, etc., where each of them have been followed by side effects and or they were not cost-effective [**3**]. Hence, finding a simple, cheap, and secure method to reduce body fat has been and is an ideal for many researchers. 

Sport activities and exercises are deemed as one of the paramount techniques [**21**], which have been drawn attention to by many researchers. 

However, the exercise kind and the intensity may make us achieve this goal, which has made the researcher examine various the exercise volume and their impact on the reduction of fat, especially body fat. 

It seems that the endurance and aerobic exercises can achieve a better position than the other exercises in this regard. 

The endurance exercise increase the maximum consumed oxygen (Vol. 2 Max) and also improve the capability of skeletal muscles to produce energy via the aerobic system [**1**]. The endurance exercises also reduce weight and increase the aerobic potential [**2**]. 

Today, the Neural-Muscular Electric Stimulation (NMES) is also proposed as another effective technique to reduce the lipid volume (skin fold) particularly in the topical form, to the amount that the body fitness institutes have tried to utilize electrical currents as a model for the quick building of body fitness and the creation of an appropriate style without benefitting from an active exercise plan for the persons who lack the adequate time for doing exercise plans [**23**]. 

In the present study, which was carried out on healthy females of an age range (20-40 years), the impact of 2 methods of aerobic training and electrical stimulation on the decreasing of abdominal fat was examined and their impacts related to each other. Given this fact, the impact of electrical stimulation and aerobic training on reduced body fat has been separately studied in the previous investigations; therefore, the present research were carried out to evaluate the impact of these two methods on the reducing of abdominal fat. 

## Materials and methods

The research community in the current research involved of 60 qualified volunteers, who have participated in this investigation. The invitation notice was published in Milad (Birth) Weekly in Lar Town to partake in this research. The qualifications for entering this study included age (20-40 years), rate 20≤BMI≤30, lack of any certain disease like cardiovascular, diabetes, thyroid hypo- and hyperactivity diseases, metabolic and hormonal disorders, not using certain drugs, lack of certain nutrient diet, not smoking, lack of regular exercises over the previous six months, without a pregnancy history more than two times and also premature childbirth before 6 months. The condition for the exclusion from this test was the absence in one exercise session as well as the absence of their interest in resuming the participation in the test. 

60 participants enrolled in this test by receiving this invitation notice so the weight and height of whole of them calculaed and their BMI was calculated. 45 participants with the BMI rate (20-30) selected and the rest 15 persons were excluded because of a much ratio of BMI. A briefing session was held for the elected participants and all these persons were informed about the research goals and conditions. 5 persons have declared their rejection regarding the participation in this study after being informed of the analysis states and the rest 40 participants were randomly classified into two Electrical Stimulation Group (ESG) and Aerobic Group AG (20 members per group). During the test outcomes, 2 persons from the ESG group and 1 person from the AG group were excluded from this test because of their absence in these sessions. 

**Measurement tools**

OMRON standard scale (Germany made) with the accuracy ratio of 0.1 kg per a kilogram was utilized to measure weight and it calibrated with one-kilogram weight after every 10-time weighting. The subcutaneous lipoma (fat) calculated by skin-fold calipers (Ponderal Model, Germany made). Crino reported the iteration and estimation of the fat skin-fold in several issues of the body as 0.95 by this device and its measurement error being of 0.8-1 mm. The subcutaneous lipoma was measured by skin-fold calipers (Ponderal Model, Germany made) in standing mode in 7 points of the body of participants including one inch distant from the right side of the navel (umbilical cord) vertically, in the point of the ilium crest, a little forward and above it obliquely, the middle among the patella and the skin-fold of groin vertically, the sub scapular area obliquely, just under the lower angle of the shoulder with an angle of 45° with regard to the horizontal axis toward the central line, behind the arm, at the middle among the bone prominence (acromion) and the elbow lump under the condition that the hand is placed open and easily beside the body, the thoracic area (chest) obliquely in a point at a distance of one-third of the line in the anterior armpit and tip of chest and in the armpit area, vertically, on the middle line of the armpit, at the level of the chest tip. The measurement was started from the right position of body after marking the given area with a black magic pen, this being done twice for a higher accuracy and if there was more than one-millimeter difference among the two measurements, so, the measurement was performed for the third time and the average of the 2 closer sizes was recorded as subcutaneous lipoma index. The fat measurement test was not done twice immediately hence that the skin-fold did not exit its natural mode. 

Also height calculated via the Seca height scale with 1-mm precision rate that influenced via the minimum ambient impacts. Therefore, it could be implied that this tool could enjoy the needed reliability. 

To measure BMI, the weight (Kg) to square height (m) ratio formula was employed [**7**]. 

Jackson-Pollock formula utilized to compute fat percentage and fat-free mass of body and initially the skin-fold of subcutaneous lipoma was estimated in 7 body points and at the second step density (concentration) of body of the given participant it was calculated and the finally fat percentage was acquired. 

To control the intensity of exercise during walking, the radial heart rate of the individual was personally evaluated and the heat rate monitor (Polar Electro model, Finland) was also utilized for this purpose and the Karvenon formula utilized to evaluate the intensity of aerobic exercises. 

The 6-channel Beautistim Stimulator (680B model, Isfahan Novin Medical Engineering Company) utilized to test the electrical stimulation group, which was calibrated via one of the medical engineers in Lar town every week. To decrease the skin-fold strength, the given area was cleaned with alcohol and cotton gauze before employing the electricity current [**22**]. 

**Findings**

The information of the collected data was shown in 3 tables after the statistical analysis and in order to express the research findings and the acquisition of its objectives. 

Primarily, normality of data distribution was analyzed by means of Kolmogorov-Smirnov test and after the confirmation of the equality of variances and normality of data; the parametric techniques were adapted for data analysis. To describe data, parameters of mean and standard deviation and pairwise t-test were used for intergroup comparison and a free t-test was employed to evaluate the mean of parameters in the two groups. 

The demographic attributes of participants are presented in **Table 1**. Two teams related to each other regarding parameters like age, weight, BMI, fat percentage, and fat-free body mass and the homogeneity of the 2 teams verified via Kolmogorov-Smirnov test. 

As it observed in **Table 1**, there is no clear distinct in the two tested groups regarding age, weight, BMI, fat percentage, and fat-free body mass (p≥0.001), so, this indicates a normal distribution of data in 2 teams.

The values of weight, BMI, fat percentage, fat-free body mass prior and later testing the 2 teams is presented in **Table 2**. With respect to information in the table, it was seen that the studied indices including weight, BMI, and body fat percentage in both groups are clearly decreased (p≤0.001) and the rate of the fat-free body mass is clearly advanced in 2 teams (p≤0.001). 

**Table 1:** Mean and standard deviation of indices before testing in the investigated teams

Attribute	Studied group		Significance level
	Electrical stimulation (18 participants)	Walking (19 participants)	
	Mean ± standard deviation	Mean ± standard deviation	
Age (years)	29.5 ± 4.53	30.26 ± 5.37	0.258
Weight (kilograms)	64.72 ± 7.13	65.95 ± 8.18	0.870
Mass Body Index (kg/ m²)	26.11 ± 2.66	26.14 ± 2.18	0.241
Body fat (percent)	38.11 ± 3.66	38.03 ± 3.35	0.882
* It is significant at level (p≤0.001); ** It is significant at level (p≤0.05)			

**Table 2:** Values of the studied variables prior and later test in 2 teams

Variable	Group	Time	Mean ± standard deviation	Mean difference	Degree of freedom	T-value	Significance level
Weight (kg)	Electrical stimulation	Before	64.72 ± 7.13	1.15	17	5.30	0.000 *
		After	73.57 ± 6.86				
	Walking	Before	65.95 ± 8.18	1.62	18	4.26	0.000 *
		After	64.33 ± 7.37				
Body mass index (kg/ m²)	Electrical stimulation	Before	26.11 ± 2.66	0.56	17	6.42	0.000 *
		After	25.54 ± 2.61				
	Walking	Before	26.14 ± 2.18	0.71	18	4.83	0.000 *
		After	42.25 ± 2.01				
Body fat (%)	Electrical stimulation	Before	38.11 ± 3.66	7.28	17	14.20	0.000 *
		After	30.82 ± 3.71				
	Walking	Before	38.03 ± 3.35	7.33	18	14.14	0.000 *
		After	30.70 ± 3.48				
Fat-free body mass (%)	Electrical stimulation	Before	39.95 ± 4.08	3.87	17	12.05	0.000 *
		After	43.83 ± 3.83				
	Walking	Before	40.75 ± 4.40	-2.64	18	-2.24	0.037 **
		After	43.40 ± 5.30				
* It is significant at level (p≤0.001); ** It is significant at level (p≤0.05)							

**Table 3:** Comparison of variables between two groups of aerobic training and electrical motivation

Variable	Group	Mean ± standard deviation	Degree of freedom	T-value	Significance level
Weight (kg)	Electrical stimulation	63.57 ± 6.86	35	-.0324	0.807
	Walking	64.33 ± 7.37	34.99	-0.324	
Body mass index (kg/ m²)	Electrical stimulation	25.54 ± 2.62	35	0.154	0.155
	Walking	25.42 ± 2.01	31.86	0.153	
Body fat (%)	Electrical stimulation	30.82 ± 3.71	35	0.108	0.847
	Walking	30.70 ± 3.48	34.50	0.108	
Fat-free body mass (%)	Electrical stimulation	43.83 ± 3.83	35	0.283	0.177
	Walking	43.40 ± 5.30	32.76	0.286	

The values of weight, BMI, fat percentage, and percentage of fat free body mass later the test in two teams are shown in **Table 3**. With respect to the above table, it was seen that after conducting this study, there is no clear distinct whiten the amounts of these indices among the 2 tested teams (p≥0.001). 

## Discussion 

The results of the independent t-test indicated that the tested persons weight in both groups was clearly decreased later eight weeks of walking and the electrical stimulation in abdominal area of their bodies (p≤0.001). In fact, the mean weight has been reduced in the groups of walking and the electrical stimulation from 65.95 ± 8.18 and 64.72 ± 7.13 to 64.33 ± 7.37 and 63.57 ± 6.86 respectively. In the other hand, 1.62 kg and 1.15 kg reduction of weights were seen respectively in walking and electrical motivation teams. The outcomes of the current research are in agreement via the results of investigation done by Hayati (2011) while they were not aligned with the findings from studies of Damirchi (2008), Porkari (2002), and Porkari (2004). It obvious that the cause for such a consistency was due to age, gender, body mass, type and intensity of stimulation plan, while the reason for the misalignment with the aforesaid studies can probably refer to the type of the electrical stimulation device, period of stimulation, methodology, and the tested persons age and especially the rats applied as tested persons in the survey of Damirchi. 

In 1948, Hawkins declared for the initial time that the electric motivation of the abdomen increases calories with a repeated regulation of a natural weight control center, stimulation of Aα and Aβ fibers, effect on obesity rate and as a finding a rising of metabolism of the given tissue [**5**]. 

Aerobic exercise also affects the body weight loss because of the advance in the energy provided by lipolysis [**20**]. 

The statistical analysis showed that BMI is clearly decreased later eight weeks of walking and 8 weeks of electrical stimulation of the abdomen (p≤0.001). Namely, both walking and the electrical motivation might reduce BMI. The independent T-test was employed to specify the clear distinct between the 2 teams in that, the sequences determined that there is no vital distinction amongst 2 teams (p = 0.155). 

The findings of the current research were consistent with the findings of investigations of Habibzadeh (2010) and Miatic (2002), while they were not aligned via the outcomes of Porkari (2002) and Porkari (2004), who claimed that the electrical motivation in the abdominal field did not affect the BMI. Probably the kind of stimulator device, the tested persons age, period of stimulation, and way of execution of test might be assumed as reasons for such a difference. 

The reason for the decrease of BMI may be justified this way; both the aerobic exercise and the electrical stimulation caused a weight loss in the present investigation. 

Thus, with regard to the formula for calculation of BMI *(BMI = Weight/ Square height)*, as BMI is decreased, the weight would more be reduced. 

Body fat percentage is clearly decreased in the current research (p≤0.001). The findings of the current research are consistent with the results of the studies of Habibzadeh (2010), Miatic (2002), Nikpoor (2008), and Damirchi (2008) while they are not aligned with the findings of the survey by Park (2003). The selected BMI, type of executed exercise and intensity and period of exercise were probably considered the reasons for the inconsistency with the priory refered study. 

The muscles electrical motivation in the abdominal area might probably lead to the consumption of the additional calories and the reduction of fat percentage by passive exercise. 

Vermiform contractions and muscular longitudinal contractions affected via the application of electrical stimulation resulted in the contraction of muscles and displacement of intercellular fluid and they partake to the omission of metabolites. Therefore, the better conditions were prepared for the omission of metabolites by the opening of the capillaries, which were at relaxation and closed status and this trend led to the advancement of transferring blood to the given area. This trend lead to an enhancement of the muscular mass. Doing aerobic exercises increases the usage of lipids as a fuel during exercises. The low- and average intensity aerobic exercises cause a further consuming of lipids as the energy source and this will cause a decrease of body fat per se [**4**]. Horton and Bravan (2001) declared that our body needs to be permanently active all over the period of exercise in order to burn calories and walking is a continuous and gradual activity that efficiently causes lipids burning [**11**]. 

Likewise, the estrogen hormone in females increases the speed of lipid metabolism in women, especially during the aerobic exercises with a rising of blood stream current in the tissue of adipose. As a result, the mutual effect of epinephrine and Estrogen Receptor β (ER-β) increases the rate of transferring free fatty acids from adipose tissue to active muscles in tissue of adipose, being improved during exercises [**6**]. 

## Conclusion

The findings of the current research showed that walking with the intensity of a stored heart rate (50-70%) might significantly cause weight loss and it can be used as an efficient, secure, and cheap strategy in reducing body fat and in preventing obesity. Similarly, by regard to this important point, there is a social class of population in the community composed of old persons and the society of disabled and cases with osteal traumas and lesions, that they cannot walk and they seek to find a method to reduce body fat, thus the electrical stimulation in the abdominal area can be employed to achieve this objective. 

**Acknowledgements **

We hereby state our thankful to the respected personnel in Mehr Physiotherapy Center at Larestan Town as well as the honored principal of Al-Zahra Primary School of Lar town and all the dear friends, who assisted us in implementing this study. 
